# Digitalization of circulation and sustainable resilience of food systems: spatial effects and transmission mechanisms

**DOI:** 10.3389/fnut.2026.1743866

**Published:** 2026-03-16

**Authors:** Hengli Wang, Qi Wang, Xinpeng Cao, Zuojunli Zhang

**Affiliations:** 1 Wuhan University of Cyber Security Preparatory Office, Wuhan, China; 2 School of Mathematics and Statistics, Zhongnan University of Economics and Law, Wuhan, China; 3 Wenlan School of Business, Zhongnan University of Economics and Law, Wuhan, China; 4 Independent Researcher, Wuhan, China

**Keywords:** digital circulation, factor market distortions, food security, food systems, industrial convergence, sustainable resilience

## Abstract

In the context of increasing global challenges to food security, including climate change, economic disruptions, and supply chain instability, the digital transformation of agricultural circulation systems has attracted growing attention as a potential driver of sustainability and resilience. Using panel data from 30 Chinese provinces spanning 2013–2022, this study empirically examines the impact of digital transformation of circulation (DTC) on the sustainable resilience of food systems (SRFS). A fixed-effects model is employed, complemented by spatial econometric models, mediating effect analysis, and threshold models to capture spatial spillovers, underlying mechanisms, and nonlinear effects. The results indicate that DTC significantly enhances SRFS by optimizing the structure of the food industry, and these findings remain robust after addressing endogeneity and conducting sensitivity analyses. Spatial analysis reveals pronounced heterogeneity in spillover effects: DTC exerts a negative indirect effect on geographically adjacent regions, while generating positive spillovers for regions with strong agricultural trade linkages. Mechanism analysis shows that these effects are driven by the alleviation of factor market distortions and the promotion of industrial convergence. Furthermore, the impact of circulation digitalization on SRFS exhibits diminishing marginal effects and a threshold effect associated with agricultural industrial agglomeration. Taken together, the findings underscore the importance of circulation digitalization in strengthening food system resilience and provide empirical support for the design of more targeted and differentiated digital development strategies in agriculture.

## 1 Introduction

Facing the combined impact of multiple external risks such as extreme climate events, geopolitical conflicts, and economic recessions (1), enhancing the sustainability of food systems has become a global consensus and an urgent need (2). The outbreak of COVID-19 has further intensified national concerns regarding food security and supply stability (3). A stable food supply constitutes not only the foundation for ensuring adequate and balanced nutrition, but also a critical prerequisite for mitigating survival risks arising from sudden shocks, including natural disasters and price volatility. At the same time, the agri-food system represents one of the major contributors to global climate change and air pollution (4). According to statistics, approximately one-third of global anthropogenic greenhouse gas emissions originate from food-related activities (5), posing substantial challenges to environmental sustainability and biodiversity conservation. Therefore, building a sustainable food system has become an integral component of the United Nations Sustainable Development Goals and a widely recognized pathway for addressing multiple global risks.

The Food and Agriculture Organization of the United Nations (FAO) defines a sustainable food system as one that ensures long-term food security while accounting for economic, social, and environmental sustainability. In developing countries such as China, traditional production and circulation systems dominated by smallholder farming are often characterized by low efficiency and weak coordination across different stages, which constrains the enhancement of sustainable resilience of the food system (SRFS). As an important link between agricultural producers and consumers (6), digital transformation of the circulation (DTC) can improve the efficiency of agricultural product distribution, strengthen linkages along the agricultural supply and industrial chains, and facilitate more coordinated system operation. From this perspective, DTC provides a relevant analytical lens for examining pathways to enhance SRFS.

Existing studies have identified multiple factors influencing either the economic resilience of the food system or the ecological performance of agricultural production. From the economic perspective, inclusive finance (7), mechanization levels (8), and agricultural insurance (9) have been shown to enhance agricultural risk resistance and income stability. From the ecological dimension, green finance (10), land circulation (11), government and market incentives (12), and Internet penetration (13) contribute to green agricultural development. In addition, environmental regulation serves as an important policy instrument for promoting ecological protection, green technological transformation, and industrial structure upgrading (14), while also playing a role in safeguarding food security (15).

While these studies provide valuable insights, SRFS inherently reflects the joint performance of economic resilience and ecological sustainability. Existing research has tended to examine these dimensions separately, with relatively limited attention to their interaction, as well as to the roles of rural wellbeing and government governance in sustaining labor supply and coordinating the food system.

In parallel, a growing body of research has examined the economic consequences of DTC. Empirical evidence suggests that the development of the circulation sector can help narrow the urban–rural income gap (16), while enhanced digitalization improves the efficiency of natural resource utilization (17) and strengthens the linkage between transaction cost reduction and value creation (18). As a key channel for allocating agricultural production factors and facilitating the exchange of agricultural products, the circulation industry exerts a systemic influence on the spatial distribution of supply and demand. Its digital transformation may contribute to alleviating regional mismatches, reducing resource overexploitation and waste, and promoting a more coordinated balance between economic and ecological outcomes within the food system. However, existing studies have rarely examined the DTC and SRFS within a unified analytical framework, particularly with respect to their structural linkages and spatial interactions.

**Figure 1:**
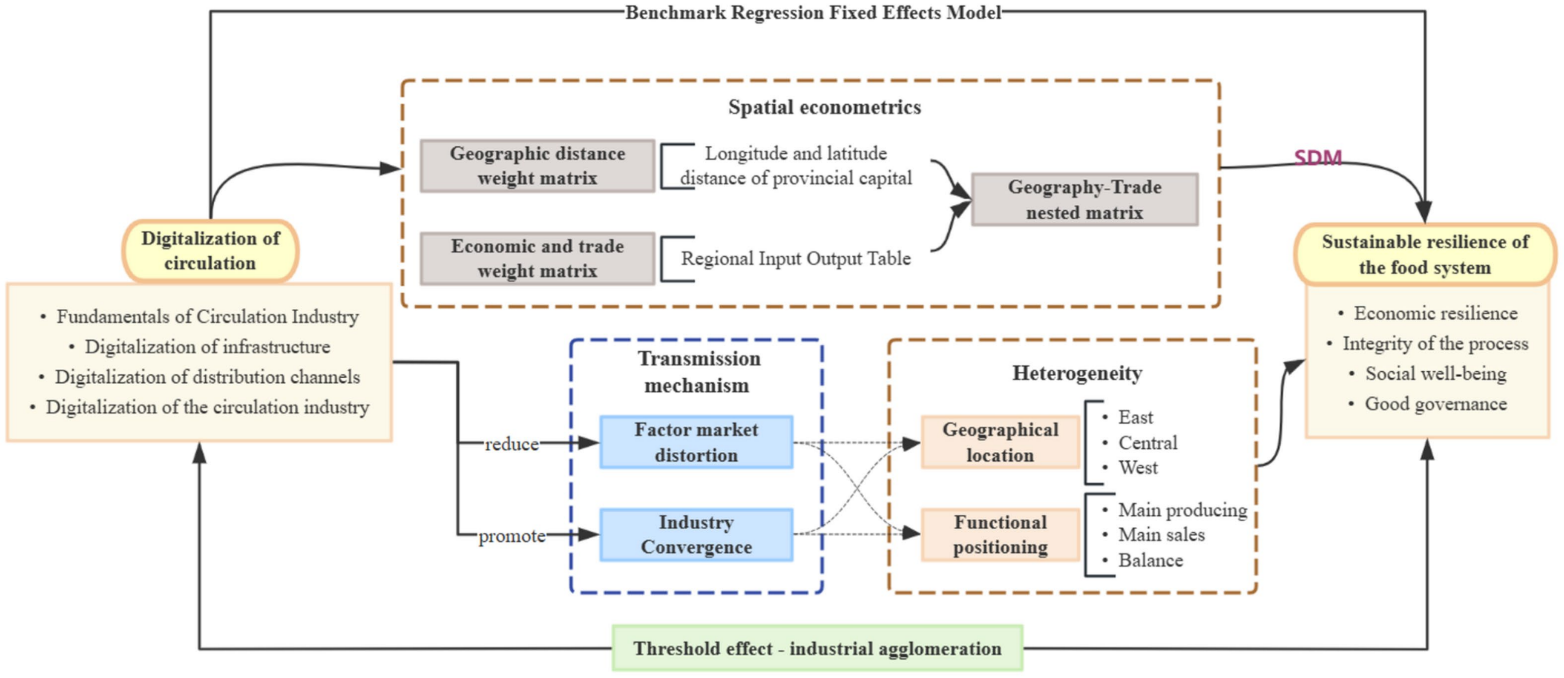
Research methodology framework.

To address these issues, this study employs a fixed-effects model based on panel data from 30 Chinese provinces over the past decade to examine the relationship between DTC and SRFS. The analytical framework is presented in Figure 1.

The contributions of this study are threefold. First, the dependent variable is extended to capture both economic resilience and environmental sustainability, while explicitly incorporating the role of multiple stakeholders within the food system. Second, the study analyzes the economic consequences of DTC for SRFS by exploring mechanisms such as the alleviation of factor market distortions and industrial convergence. Third, an interprovincial economic trade matrix is constructed using input–output tables of agricultural products across regions, enabling a more interpretable assessment of the spatial spillover effects of circulation digitalization from the perspective of trade interactions.

The remainder of this article is organized as follows. Section 2 presents the research hypotheses. Section 3 describes the study design. Section 4 reports the benchmark regression results and robustness tests. Section 5 provides an in-depth analysis of spatial effects, transmission mechanisms, and threshold effects. Section 6 concludes with discussion and policy implications.

## 2 Theoretical framework and research hypotheses

**Figure 2:**
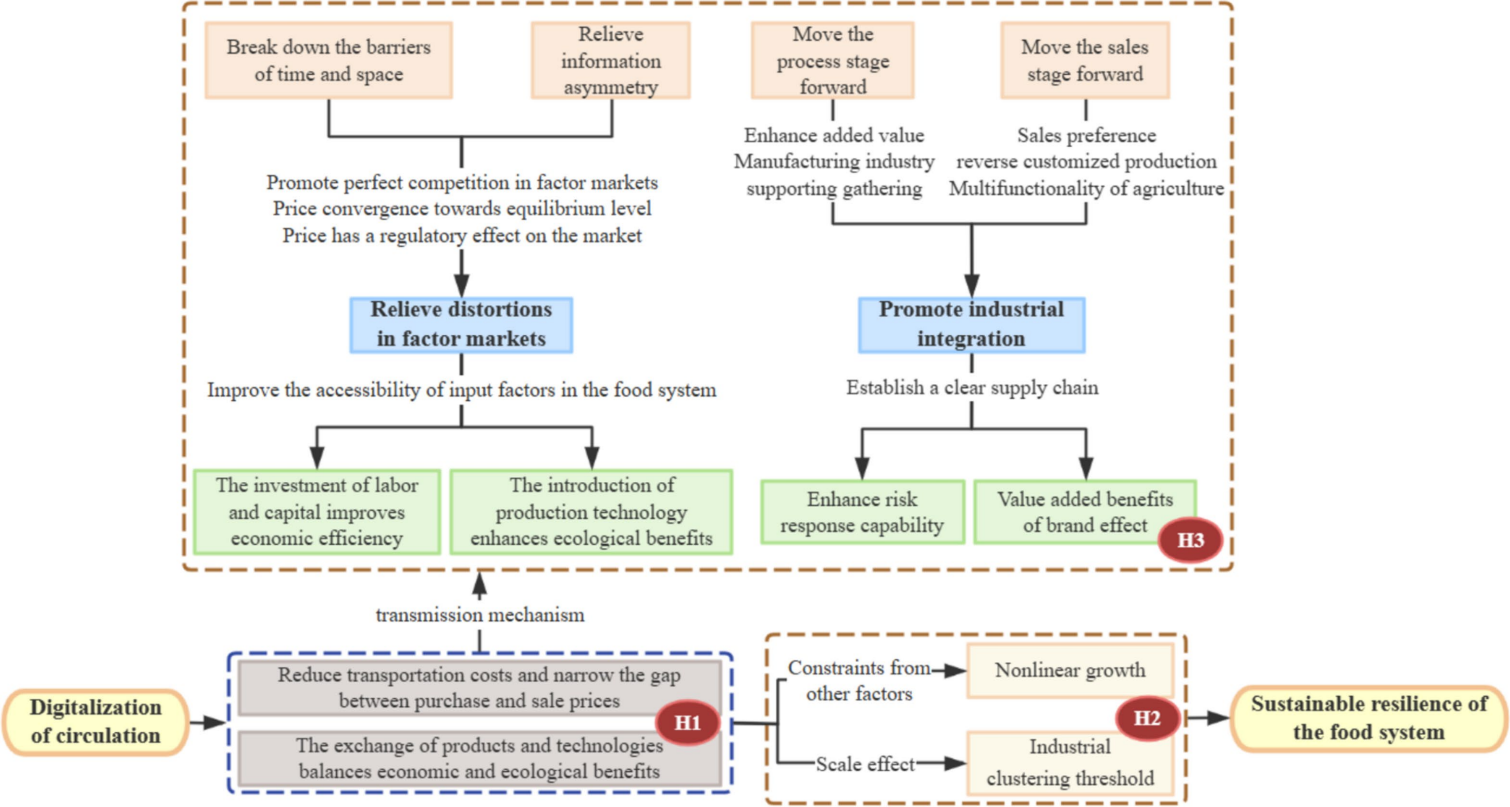
Hypothesis theoretical analysis framework.

To better understand how DTC influences the SRFS, this section develops a theoretical framework and proposes corresponding hypotheses as shown in Figure 2. Drawing on transaction cost theory, factor allocation theory, and agglomeration economics within an economic–ecological perspective, we identify three key mechanisms: (1) the overall effect of circulation digitalization on SRFS; (2) its mediating effects through alleviating factor market distortions and promoting industrial convergence; and (3) a potential nonlinear relationship shaped by the level of industrial agglomeration. These mechanisms provide the theoretical basis for the following research hypotheses.

The traditional grain circulation pattern mainly relies on intermediaries purchasing grain from farmers at relatively low prices and selling it to end consumers at higher prices. Such a pattern generates substantial inefficiencies, including the excessive depletion of labor, capital, and technological resources (19), thereby constraining both economic and ecological sustainability. The underlying cause lies in persistently high transportation and transaction costs, which circulation digitalization has the potential to alleviate (20). Reductions in agricultural trade costs are conducive to improving agricultural production levels and stimulating the GDP within agricultural and agri-food systems (21). In underdeveloped circulation systems, inefficiencies in the flow of production factors and products tend to persist (20). By enhancing circulation efficiency, improving information accuracy (22), and fostering more direct connections between farmers and consumers, circulation digitalization facilitates urban–rural integration (23). Through technology sharing and product exchange, digitalization contributes to a more coordinated alignment between economic and ecological objectives, thereby supporting improvements in SRFS.

*Hypothesis 1*: DTC enhances SRFS by improving supply chain efficiency, reducing transaction costs, and facilitating information flows, thereby strengthening adaptability to climate change, economic disruptions, and other external shocks.

The circulation industry functions as an intermediary that transforms production factors into goods and services (6). Its digital transformation relaxes spatial and temporal constraints, reduces information asymmetry (24), and improves market efficiency, contributing to a more balanced interaction between supply and demand. As a result, factor market distortions are alleviated, and the availability of key inputs—such as labor, capital, and technology—within food systems is enhanced. On the one hand, sufficient labor and capital inputs help raise agricultural total factor productivity (25); on the other hand, the adoption of green production technologies mitigates the environmental impacts of agriculture, contributing to the coordination of economic and ecological objectives (52).

At the same time, circulation digitalization enables new sales models, including e-commerce, which promotes disintermediation and increases the value added of agricultural products. The forward extension of processing stages enhances value creation and attracts the agglomeration of related manufacturing industries. Meanwhile, the forward shift of sales stages generates a reverse customization effect of consumer preferences on production, accompanied by the multifunctionality of agriculture (27), thereby fostering closer integration between agriculture and secondary and tertiary industries. This process of industrial convergence strengthens linkages among production, processing, and sales within the food system, contributing to a more coherent agricultural supply chain. In turn, it enhances risk-coping capacity and brand value creation (28), while easing the tension between smallholder-based production and large-scale market demand in developing countries (29).

*Hypothesis 2*: DTC enhances SRFS by alleviating factor market distortions and promoting industrial convergence, thereby facilitating more efficient integration of agriculture with manufacturing and services and strengthening both economic and ecological resilience.

The law of diminishing marginal utility suggests that, beyond a certain level, further digitalization may yield decreasing marginal effects on SRFS, while other constraints—such as climate risks—become increasingly salient (30). Accordingly, the relationship between DTC and SRFS may exhibit nonlinear characteristics (31).

Industrial agglomeration, which supports regional economic development through increasing returns to scale and knowledge and technology spillovers, may condition the effectiveness of circulation digitalization in enhancing SRFS (32). When the level of agricultural industrial agglomeration is relatively low, synergistic effects among resource factors are difficult to fully realize, and digital dividends in the circulation sector are less likely to translate into effective spillover effects due to an insufficient industrial base (33). As industrial agglomeration surpasses a critical threshold, the spatial concentration of labor and resources can substantially reduce the diffusion cost of digital technologies, enabling more efficient integration of logistics, information flows, and capital flows (34). This process provides a stronger industrial foundation and a more collaborative environment for circulation digitalization.

*Hypothesis 3*: The impact of DTC on SRFS exhibits diminishing marginal utility and may be subject to a threshold effect associated with industrial agglomeration, beyond which digital technology adoption becomes more efficient and contributes more strongly to food system resilience.

## 3 Materials and methods

### 3.1 Data and variables

#### 3.1.1 Variable measurement

##### 3.1.1.1 Dependent variable

Sustainable Resilience of Food Systems (SRFS): In this study, SRFS refers to the capacity of food systems to maintain stability, adapt to shocks, and sustain ecological, economic, and social performance over time. Drawing on FAO’s definition of food system sustainability (35), an evaluation system for SRFS is constructed by selecting 24 indicators across four dimensions: economic resilience, environmental sustainability, social wellbeing, and good governance. These dimensions collectively reflect the economic inclusiveness, ecological balance, social equity, and institutional adaptability required to achieve long-term food security under global challenges such as climate change, economic disruptions, and resource constraints. The entropy method—chosen for its objectivity in determining indicator weights based on data dispersion—is applied to allocate weights to each indicator, and the SRFS level is calculated for each province and year (as shown in Table 1). The 24 indicators are derived from FAO’s sustainability framework and adapted to the Chinese context, ensuring that both the roles of key system participants (e.g., farmers, governments) and the multidimensional nature of food system resilience are fully represented.

**Table 1:** SRFS system and weights.

Dimension	Indicator layer	Attributes	Weights
Economic resilience	Total power of agricultural machinery per unit area	+	0.0346696
	Effective irrigation rate	+	0.0349459
	Grain yield per unit area	+	0.0269836
	Proportion of output value of agriculture, forestry, animal husbandry, and fishery service industries	+	0.1563506
	Agricultural labor productivity	+	0.0272504
	Agricultural product producer price index	+	0.0091877
Environmental integrity	Crop failure rate	−	0.0020414
	Fertilizer application per unit area	−	0.0121519
	Pesticide application per unit area	−	0.0024278
	Installed area of solar water heaters in rural areas	+	0.075345
	Per capita electricity consumption in rural areas	+	0.172788
Social wellbeing	Per capita expenditure on education, culture, and entertainment in rural areas	+	0.0122954
	Number of cultural stations in townships	+	0.0533297
	Coverage rate of TV programs in rural areas	+	0.0052504
	Number of computers per hundred households in rural areas	+	0.0517405
	Engel’s coefficient of rural resident families	−	0.005221
	Per capita disposable income of rural resident families	+	0.0274564
	Per capita housing area	+	0.0297548
	Number of air conditioners per hundred households in rural areas	+	0.0807794
Good governance	Proportion of rural residents receiving minimum living security	−	0.0080701
	Average number of village committees per ten thousand people	+	0.039969
	Number of health professionals per thousand people	+	0.0329436
	Proportion of villages with sewage treatment	+	0.0750992
	Road pavement rate	+	0.0239486

##### 3.1.1.2 Independent variable

Digital Transformation of the Circulation (DTC): Previous studies suggest that the digitalization of circulation includes three key dimensions: digital infrastructure, digitalization of circulation channels, and the digitalization of the circulation industry (36). Recognizing the need for a more comprehensive perspective, this study introduces the “foundation of the circulation industry,” which encompasses the existing infrastructure and technological capabilities critical for digital integration. Based on this framework, we select 11 indicators to construct an evaluation system for the level of DTC (as shown in Table 2). DTC refers to the integration of digital technologies across supply chains, logistics, market platforms, and stakeholder networks. These changes improve the efficiency, transparency, and sustainability of food systems, enhancing their resilience to climate change, economic disruptions, and other external shocks. The 11 indicators selected for DTC cover areas such as e-commerce adoption, logistics efficiency, digital communication networks, and technology integration across farmers, governments, and markets. These indicators were chosen based on existing literature and global frameworks on digitalization in food systems (36), with a focus on core components of digital transformation: infrastructure, digital platforms, and industry integration.

**Table 2:** DTC system and weights.

Dimension	Indicator layer	Attributes	Weights
Foundation of the circulation industry	Per capita postal and telecommunications business volume	+	0.1733567
	Proportion of employment in the circulation industry	+	0.1552347
	Share of the circulation industry’s output value in regional GDP	+	0.0221762
Digitalization of infrastructure	Internet broadband access ports	+	0.0832435
	Length of optical cable lines	+	0.0898356
	Mobile phone penetration rate	+	0.038591
Digitalization of distribution channels	Share of e-commerce sales in regional GDP	+	0.1055079
	Proportion of enterprises with e-commerce transactions in the total number of enterprises	+	0.0383131
	Wholesale and retail coefficient	−	0.0063635
Digitalization of the circulation industry	Per capita express delivery quantity	+	0.2382773
	Depth of digital finance usage	+	0.0491006

##### 3.1.1.3 Mechanism variables

Based on the literature review in Section 2 and the mechanism hypothesis (H2), this study defines the degree of factor market distortion (FMD) and the level of industrial convergence (IC) as two main transmission mechanism variables, with specific definitions as follows. Both mechanisms are closely tied to the structural transformation of food systems. Reducing factor market distortions enhances the efficiency of agricultural input allocation, contributing to economic resilience and equitable resource distribution. Meanwhile, industrial convergence strengthens vertical integration and value-chain coordination, which are essential for building adaptive and sustainable food systems under climate and market shocks.

###### 3.1.1.3.1 Factor market distortion (FMD)

This study defines the relative gap between the development of factor markets in each province and the maximum value in the sample as a proxy variable for FMD (37). The specific calculation method is shown in Equation 1:


$$\mathrm{FM}D_{i}=\frac{\max({\text{factor}}_{i})-{\text{factor}}_{i}}{\max({\text{factor}}_{i})}$$ (1)

In Equation 1, $\text{facto}r_{i}$ denotes the index of factor market development for region *i* in year *t*, sourced from the *China Provincial Marketization Index Report (2021)*, covering the period from 1997 to 2019. Missing data for 2020–2022 are extrapolated based on the average growth rate of each province from 2013 to 2019. In the context of food systems, factor market distortions—such as unequal access to land, credit, and labor—reduce agricultural efficiency and hinder the resilience of production and circulation systems. Thus, lower distortion levels indicate more equitable and efficient factor allocation, fostering greater sustainable resilience.

###### 3.1.1.3.2 Industry convergence (IC)

This study employs the three-system coupling coordination model to measure the convergence level of the primary, secondary, and tertiary industries in each province. This model comprehensively reflects the coupling and coordination among the three major industries (38), as shown in Equations 2–4:


$$C_{i}=\sqrt{2-\frac{3\times (N{Y_{i}}^{2}+G{Y_{i}}^{2}+F{W_{i}}^{2})}{{\left(NY_{i}+GY_{i}+FW_{i}\right)}^{2}}}$$ (2)

$$T_{i}=\alpha_{1}NY_{i}+\alpha_{2}GY_{i}+\alpha_{3}FW_{i}$$ (3)

$$IC_{i}=\sqrt{C_{i}\times T_{i}}$$ (4)

In Equations 2, 3, $NY_{i}$ , $GY_{i}$ , and $FW_{i}$ represent the ratios of regional gross product to employment in the primary, secondary, and tertiary industries, respectively, for each region. These ratios capture the development level and productivity of the three industries. $C_{i}$ denotes the coupling degree, $T_{i}$ represents the coordination degree, and $IC_{i}$ indicates the coupling–coordination degree of the three major industries. The undetermined coefficients $\alpha_{1},$ $\alpha_{2}$ , and $\alpha_{3}$ represent the importance of the three industries, and are set to 1/3, as all three subsystems are equally significant in driving coordination within the broader food system.

In the context of food systems, industrial convergence reflects the integration of agriculture with processing, logistics, and services, promoting value-chain efficiency, reducing waste, and enhancing adaptability to external shocks. As such, a higher degree of convergence signifies a more resilient and sustainable food system.

##### 3.1.1.4 Control variables

To account for potential influences from other factors and referring to existing studies, this study controls for the following variables:

Human Capital in the Food System: Employment rate in the primary industry ( Employ ) and average years of education for rural residents ( Education ) are used as proxies for human capital (39). Human capital is critical in enhancing food system resilience, as a more educated workforce and higher employment rates in agriculture can improve the adoption and effective use of digital technologies, leading to greater productivity and resilience to market and climate shocks.

Regional Industrial Structure: Proportion of the primary industry in GDP ( Prim_prop ) measures the industrial structure of each region (48). This variable controls for the regional reliance on agriculture, as regions with a higher share of agricultural output in GDP may face greater challenges in adapting to global food system disruptions, but may also have greater potential for leveraging digital technologies to enhance food system resilience.

Agricultural Investment and Financial Service Availability: Per capita fixed asset investment in rural areas ( Invset ) and loans available to agriculture ( Loan ) are used to measure the level of financial support for the agricultural sector (41). Access to agricultural investment and financial services is critical for improving infrastructure and technological capabilities. In a digitalized circulation environment, these financial flows can be more efficiently allocated to areas of need, such as technological adoption and supply chain improvements, thereby enhancing resilience.

These control variables are important for isolating the effects of DTC on SRFS, as they account for external influences that could also shape food system resilience, such as the availability of skilled labor, financial resources, and the underlying industrial structure in each region.

##### 3.1.1.5 Threshold variables

Agricultural Industrial Agglomeration (AIA): The relative level of agricultural industrial agglomeration is measured using the concept of location entropy, which reflects the spatial concentration of agricultural production relative to the national total. This method captures special cases, such as regions with high agricultural concentration despite smaller agricultural scales, which can significantly influence food system resilience. The relative level of agricultural industrial agglomeration is measured based on the concept of location entropy using Equation 5 (42), which can accurately reflect special cases such as higher location entropy values in regions with smaller agricultural scales.


$$\mathrm{AI}A_{i,t}=\frac{a_{i,t}/\sum_{i}a_{i,t}}{\mathrm{gd}p_{i,t}/\sum_{i}\mathrm{gd}p_{i,t}}$$ (5)

In Equation 5, $a_{i,t}$ represents the total agricultural output value of province i in year t , $A_{i,t}=\Sigma_{i}a_{i,t}$ represents the total national agricultural output value in year t , $\mathrm{gd}p_{i,t}$ represents the gross domestic product (GDP) of province i in year t , and $\mathrm{gd}p_{i,t}=\Sigma_{i}\mathrm{gd}p_{i,t}$ represents the total national GDP.

This ratio reflects how concentrated agricultural production is in a region relative to its total output, capturing both geographical dispersion and regional concentration. Location entropy is preferred here for its ability to account for these spatial patterns, which traditional agglomeration measures might overlook.

Agricultural agglomeration plays a crucial role in enhancing SRFS, as regions with higher levels of agglomeration benefit from increased resource efficiency, digital technology adoption, and market integration, making their agricultural systems more adaptable to shocks and better able to leverage digital transformation for sustainability.

#### 3.1.2 Data sources and descriptive statistics

The data for this study are sourced from several national databases, including the China Statistical Yearbook, China Macroeconomic Database, China Rural and Agricultural Database, China Urban and Rural Construction Database, China Regional Economic Database, Tertiary Industry Database, and the Peking University Digital Inclusive Finance Index Report (2011–2022). These sources provide key data on economic output, industrial structure, agricultural investment, and digital finance, essential for analyzing food system resilience.

Panel data from 30 provinces in China from 2013 to 2022 are used. Tibet is excluded due to excessive missing data, which does not significantly affect the generalizability of the results. Missing values for other provinces are filled using linear interpolation based on each province’s average growth rate from 2013 to 2019. Sensitivity checks confirm that this method does not introduce significant bias.

Descriptive statistics for the relevant variables, including means and standard deviations, are presented in Table 3, providing insights into regional variations in key indicators of digital circulation, economic resilience, and agricultural development.

**Table 3:** Descriptive statistics.

Variables	*N*	Mean	SD	Median	Min	Max
SRFS	300	0.226	0.098	0.199	0.076	0.534
DTC	300	0.198	0.120	0.173	0.043	0.620
Education	300	8.038	0.876	7.953	5.861	12.600
Employ	300	0.378	0.121	0.398	0.080	0.614
Prim_prop	300	9.469	5.137	9.091	0.200	25.100
Invset	300	0.174	0.076	0.171	0.011	0.485
Loan	300	3.021	3.231	2.204	0.022	24.893
FMD	300	0.428	0.190	0.457	−0.000	0.902
IC	271	0.023	0.004	0.023	0.009	0.036
AIA	300	1.190	0.649	1.189	0.047	3.467

The results show that there are no extreme outliers in the variables used in this study. After introducing time dummy variables, the VIF for the explanatory variables ranges from 1.12 to 3.75, all of which are well below 5, indicating no multicollinearity issues. However, when regional dummy variables are included, the VIF ranges from 2.12 to 27.55, suggesting severe multicollinearity. As a result, a time-fixed effects model is chosen for the subsequent analysis to address these issues.

### 3.2 Econometric strategy

#### 3.2.1 Baseline estimation and robustness analysis

##### 3.2.1.1 Baseline model

To assess the impact of the digital transformation of the circulation on *SRFS*, this study constructs the following fixed-effects model:


$$\mathrm{SRF}S_{\mathrm{it}}=\beta_{0}+\beta_{1}\mathrm{DT}C_{\mathrm{it}}+\beta_{2}X_{\mathrm{it}}+\delta +\epsilon_{\mathrm{it}}$$ (6)

In Equation 6, *X* represents the control variables, *δ* denotes the fixed effects, and *ε* represents the random disturbance term. This paper employs robust standard errors and performs clustering adjustments at the regional level to mitigate potential issues of heteroskedasticity and serial correlation.

##### 3.2.1.2 Moderating effect analysis

To investigate the moderating effect between *DTC* and the level of government support for agriculture, the interaction term between *DTC* and the proportion of agricultural fiscal expenditure (*Finance*) is introduced into Equation 6, constructing the econometric model as shown in Equation 7:


$$\begin{array}{l} \mathrm{SRF}S_{\mathrm{it}}=\beta_{0}+\beta_{1}\mathrm{DT}C_{\mathrm{it}}+\beta_{2}\text{Financ}e_{\mathrm{it}}+\beta_{3}\mathrm{DT}C_{\mathrm{it}}\\ \times \text{Financ}e_{\mathrm{it}}+\beta_{4}X_{\mathrm{it}}+\epsilon_{\mathrm{it}} \end{array}$$ (7)

##### 3.2.1.3 Mediating effect model

Based on the theoretical analysis above, the DTC can enhance the SRFS by alleviating factor market distortions. This paper employs a stepwise regression method to test this transmission mechanism. The step-by-step regression model is outlined as shown in Equations 8 and 9:


$$\mathrm{FM}D_{\mathrm{it}}=\beta_{0}+\beta_{1}\mathrm{DT}C_{\mathrm{it}}+\beta_{2}X_{\mathrm{it}}+\delta +\epsilon_{\mathrm{it}}$$ (8)

$$\mathrm{SRF}S_{\mathrm{it}}=\beta_{0}+\beta_{1}\mathrm{DT}C_{\mathrm{it}}+\beta_{2}\mathrm{FM}D_{\mathrm{it}}+\beta_{3}X_{\mathrm{it}}+\delta +\epsilon_{\mathrm{it}}$$ (9)

Similarly, to test whether the DTC affects the SRFS through accelerated industrial convergence, this study construct the following mediating effect as shown in Equations 10 and 11:


$$IC_{\mathrm{it}}=\beta_{0}+\beta_{1}\mathrm{DT}C_{\mathrm{it}}+\beta_{2}X_{\mathrm{it}}+\delta +\epsilon_{\mathrm{it}}$$ (10)

$$\mathrm{SRF}S_{\mathrm{it}}=\beta_{0}+\beta_{1}\mathrm{DT}C_{\mathrm{it}}+\beta_{2}IC_{\mathrm{it}}+\beta_{3}X_{\mathrm{it}}+\delta +\epsilon_{\mathrm{it}}$$ (11)

#### 3.2.2 Spatial and nonlinear effects

Previous studies have shown that the construction of transportation infrastructure has a direct impact and spatial spillover effect on the economic output of the agricultural sector (43). Therefore, this paper introduces spatial weight matrices into the baseline model Equation 6 and use the Spatial Durbin Model (SDM) to decompose the direct and indirect effects of *DTC* in promoting SRFS , as shown in Equation 12:


$$\begin{array}{l} \mathrm{SRF}S_{\mathrm{it}}=\beta_{0}+\mathrm{\rho W}\times \mathrm{SRF}S_{\mathrm{it}}+\beta_{1}\mathrm{DT}C_{\mathrm{it}}+\rho_{1}W\\ \times \mathrm{DT}C_{\mathrm{it}}+\beta_{2}X_{\mathrm{it}}+\rho_{2}W\times X_{\mathrm{it}}+\delta +\epsilon_{\mathrm{it}} \end{array}$$ (12)

In Equation 12, W×SRFS , W×DTC , and W×X represent the spatial lag variables of the SRFS , DTC , and control variables, respectively. ρ denotes the spatial autoregressive coefficient, and W represents the spatial weight matrices.

##### 3.2.2.1 Threshold effect model

Considering DTC both as the core explanatory variable and as a threshold variable, this paper establishes the threshold effect model in Equation 13 to explore its nonlinear dynamic utility (44):


$$\begin{array}{l} \mathrm{SRF}S_{\mathrm{it}}=\beta_{0}+\beta_{1}\mathrm{DT}C_{\mathrm{it}}\times I(\mathrm{DT}C_{\mathrm{it}}\leq \omega )+\beta_{2}\mathrm{DT}C_{\mathrm{it}}\\ \times I(\mathrm{DT}C_{\mathrm{it}}>\omega )+\beta_{3}X_{\mathrm{it}}+\delta +\epsilon_{\mathrm{it}} \end{array}$$ (13)

In Equation 13, ω represents the corresponding threshold, and I(·) is the indicator function, where I(·)=1 if the condition in parentheses is true, and I(·)=0 otherwise.

Based on the theoretical analysis in the previous sections, the effect of the DTC on the SRFS also varies under different levels of agricultural industrial agglomeration. To identify this threshold, we use the degree of agricultural industrial agglomeration as the threshold variable and establish the threshold effect model as shown in Equation 14:


$$\begin{array}{l} \mathrm{SRF}S_{\mathrm{it}}=\beta_{0}+\beta_{1}\mathrm{DT}C_{\mathrm{it}}\times I(\mathrm{AI}A_{\mathrm{it}}\leq \omega )+\beta_{2}\mathrm{DT}C_{\mathrm{it}}\\ \times I(\mathrm{AI}A_{\mathrm{it}}>\omega )+\beta_{3}X_{\mathrm{it}}+\delta +\epsilon_{\mathrm{it}} \end{array}$$ (14)

## 4 Empirical analysis and results

This section presents the benchmark regression results of the impact of the *DTC* on SRFS , examines the moderating effect of government support for agriculture, and tests the robustness of the findings.

### 4.1 Baseline regression analysis

Based on the Baseline regression 1, this study sequentially adds control variables to examine the correlation between the *DTC* and SRFS under different dynamic combinations of control variables. The results are presented in Table 4, where Model (1) and Model (6) represent the results without control variables and with all control variables included.

**Table 4:** Baseline regression analysis and results.

Variables	(1)	(2)	(3)	(4)	(5)	(6)
	SRFS	SRFS	SRFS	SRFS	SRFS	SRFS
DTC	0.720***	0.504***	0.435***	0.426***	0.423***	0.412***
	(0.094)	(0.112)	(0.117)	(0.125)	(0.130)	(0.125)
Employ		−0.261***	−0.192***	−0.188***	−0.186***	−0.185***
		(0.055)	(0.066)	(0.065)	(0.065)	(0.066)
Prim_prop			−0.004**	−0.004**	−0.004**	−0.004**
			(0.002)	(0.002)	(0.002)	(0.002)
Education				0.004	0.004	0.005
				(0.008)	(0.008)	(0.008)
Invset					0.029	0.021
					(0.075)	(0.079)
Loan						0.001
						(0.003)
Constant	0.083***	0.225***	0.247***	0.214***	0.209***	0.202***
	(0.017)	(0.037)	(0.043)	(0.058)	(0.058)	(0.068)
Fe	Yes	Yes	Yes	Yes	Yes	Yes
Observations	300	300	300	300	300	300
$\text{Adjusted}\;R^{2}$	0.707	0.761	0.777	0.777	0.777	0.777

Robust standard errors in parentheses ***p < 0.01, **p < 0.05, *p < 0.1. The T-value is enclosed in parentheses.

The results in Table 4 show the following: (1) As control variables are introduced step by step, the main explanatory variable DTC remains significant. The regression coefficients and significance levels of the control variables change little, indicating that the regression results are robust. (2) In Model (6), which includes all control variables, the marginal effect of *DTC* on SRFS is 0.412 and significant at the 1% level. That is, a 1% increase in *DTC* is associated with an average 0.412% increase in SRFS . This finding preliminarily confirms the validity of Hypothesis 1. (3) The regression results of the control variables indicate that the employment rate in the primary industry and the proportion of the primary industry in GDP are negatively correlated with SRFS . This suggests that an economic structure overly reliant on the primary industry is unlikely to enhance the SRFS . The main reason is the lack of diversified industrial support in food systems, which hinders the optimization and modernization of the economic structures.

### 4.2 Moderating effect

The proportion of agricultural fiscal expenditure ( Finance ) and its interaction term with the level of circulation digitalization ( DTC×Finance ) are included in the benchmark regression to analyze the moderating effect. The estimation results of Equation 7 are presented in Table 5.

**Table 5:** Results of moderating effect.

Variables	(1)	(2)	(3)	(4)
		HighDTC	Low−Low	Low−High
	SRFS	SRFS	SRFS	SRFS
DTC	−0.508**	0.172	0.558**	0.856***
	(0.215)	(0.157)	(0.250)	(0.250)
Finance	−0.596***			
	(0.184)			
Digit×finance	4.374***			
	(0.794)			
Controls	Yes	Yes	Yes	Yes
Fe	Yes	Yes	Yes	Yes
Observations	300	117	133	49
$\text{Adjusted}\;R^{2}$	0.813	0.609	0.629	0.706

High DTC indicates DTC ≥ 0.2, Low-Low indicates DTC < 0.2 and Finance < 0.25, and Low-High indicates DTC < 0.2 and Finance ≥ 0.25, Robust standard errors in parentheses ***p < 0.01, **p < 0.05, *p < 0.1. The T-value is enclosed in parentheses.

**Figure 3:**
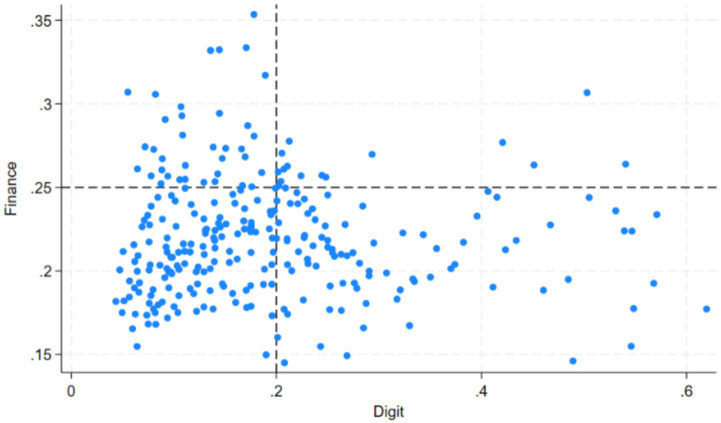
Scatter plot of Finance and DTC.

The coefficient of the interaction term between the proportion of agricultural fiscal expenditure and the level of *DTC* is significantly positive at the 1% level, with a specific estimate of 4.374. This suggests that government fiscal support plays a positive synergistic role in the process by which DTC affects SRFS . By increasing fiscal investment in agriculture, the government provides necessary infrastructure, technology introduction, and talent training in food-producing areas, thereby mitigating the urban–rural technology divide and funding issues in this process. The distribution characteristics of ( DTC , Finance ) for each sample are shown in Figure 3.

This study divides DTC into high and low levels using 0.2 as the threshold and Finance into high and low proportions using 0.25 as the threshold. This categorization results in four groups within the DTC−Finance coordinate system: highDTC−high Finance , highDTC−lowFinance , lowDTC−high Finance , and lowDTC−lowFinance . Considering the sample size of each group, the highDTC−high Finance and highDTC−lowFinance groups are combined, yielding three final groups: {( Digit , Finance ) | Digit≥0.2 }, {( Digit , Finance ) | Digit<0.2 , Finance<0.25 }, {( Digit , Finance ) | Digit<0.2 , Finance≥0.25 }. The impact of DTC on SRFS is compared across these groups, with results shown in models (1), (2), and (3) of Table 5.

The findings from Table 5 indicate that: (1) In regions with low DTC , DTC has a significantly positive effect on SRFS ; however, in regions with high DTC , the promoting effect of DTC is not significant, possibly due to the diminishing marginal utility of DTC . (2) Compared to regions with low DTC and low Finance , the promoting effect of DTC on SRFS is stronger in regions with low DTC and high Finance , further supporting the discovery of a positive moderating effect from the interaction term.

### 4.3 Endogeneity analysis and robustness test

#### 4.3.1 Endogeneity analysis

To avoid multicollinearity issues, this study does not introduce regional dummy variables in the baseline regression, which means that some factors of regional differences are not fully considered. To mitigate the endogeneity problems arising from this, this study selects the interaction term between the number of fixed telephones per hundred people in each province in 1984 (units per hundred people) and the national highway mileage from 2013 to 2022 (ten thousand kilometers) as an instrumental variable Mobil×Road (54). And then we apply two-stage least squares (2SLS) to address the endogeneity in the benchmark regression results. (1) The number of fixed telephones per hundred people in each province in 1984 reflects the initial foundation of communication facilities. It can serve as one of the historical influencing factors of circulation digitalization, satisfying the relevance requirement. (2) Since it is far removed from the study period, it cannot directly affect SRFS , thus meeting the exogeneity requirement. (3) National highway mileage is a crucial infrastructure for the circulation industry. By multiplying the number of fixed telephones per hundred people in each province in 1984 with the national highway mileage from 2013 to 2022, a time-varying factor is introduced without altering regional differences.

Model (1), (2) in Table 6 present the first-stage and second-stage results of the instrumental variable regression. The regression result of the instrumental variable indicates that after addressing the endogeneity issues, the digital transformation of the circulation continues to have a significant positive impact on SRFS . This confirms that the main conclusion of the benchmark regression in this study remains valid.

**Table 6:** Endogeneity analysis and robustness test.

Variables	(1)	(2)	(3)	(4)	(5)
	1st stage	2nd stage	2013–2017	2018–2022	Quantile regression
	DTC	SRFS	SRFS	SRFS	SRFS
Mobil×Road	0.431***				
	(0.097)				
DTC		0.623***	0.336**	0.621***	0.523***
		(0.191)	(0.137)	(0.127)	(0.080)
Fvalue	19.823***				
Controls	Yes	Yes	Yes	Yes	Yes
Fe	Yes	Yes	Yes	Yes	Yes
Observations	300	300	150	150	300
$\text{Adjusted}\;R^{2}$	0.843	0.770	0.705	0.825	0.573

Robust standard errors in parentheses ***p < 0.01, **p < 0.05, *p < 0.1. The T-value is enclosed in parentheses.

#### 4.3.2 Robustness test

In 2018, China first launched the construction of modern supply chain systems in the circulation sector, specifically targeting the circulation of agricultural products. To exclude the impact of this policy for robustness testing (45), the time span of the total sample is divided into periods before (2013–2017) and after (2018–2022) the policy was proposed. Additionally, to eliminate the influence of extreme values, quantile regression is performed based on the 50th percentile of SRFS for another robustness test. The regression results of the two methods are shown in Model (3), (4), and (5) of Table 6, where the coefficient signs and significance levels of the core explanatory variable do not change. This result further confirms the positive impact of DTC on SRFS , and the main conclusion of this study remains valid.

## 5 Expanded analysis

This section presents the benchmark regression results of the impact of the DTC on SRFS, examines the moderating effect of government support for agriculture, and tests the robustness of the findings.

### 5.1 Spatial distribution and spatiotemporal patterns

**Figure 4:**
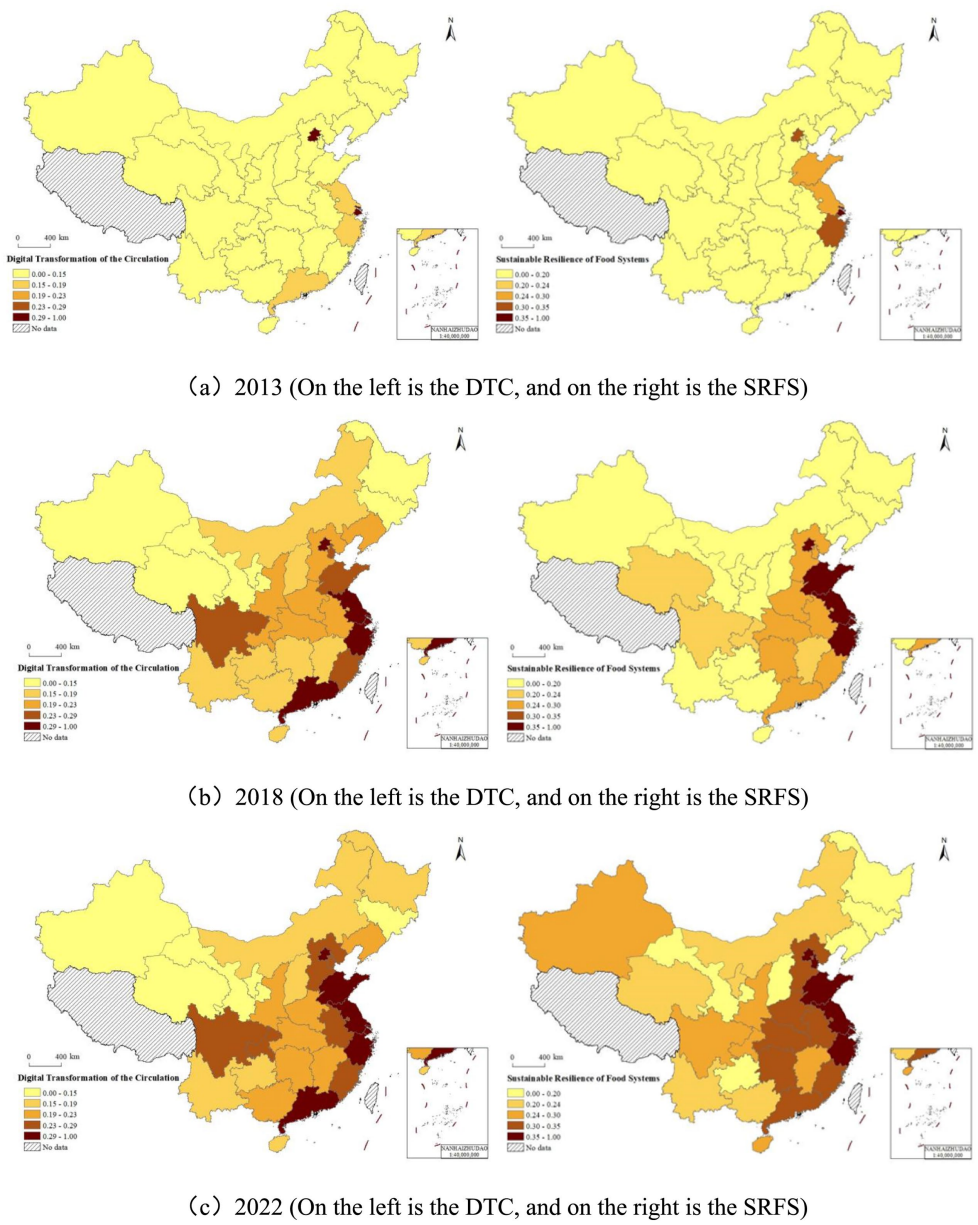
Spatial–temporal pattern of DTC and SRFS in different years: **(a)** 2013, **(b)** 2018 and **(c)** 2022. This map is based on the official standard map (review number GS (2024)0650) obtained from the Standard Map Service of the National Bureau of Surveying and Mapping Geographic Information of China. The base map has not been modified.

Figure 4 illustrates the spatial–temporal patterns of the DTC and the SRFS in 2013, 2018, and 2022: (1) In the spatial dimension, both DTC and SRFS exhibit clustering characteristics, with high-level regions concentrated in the southeast coastal areas of China, while the western inland regions are significantly lower than other areas. (2) In the temporal dimension, the high-level regions of DTC and SRFS have gradually extended from the southeast coastal areas to the central regions, and the levels of DTC and SRFS in each province have progressively increased. (3) The co-directional clustering in space and co-directional development trend over time between DTC and SRFS preliminarily indicate the robustness of the benchmark regression results and the necessity of employing a spatial model to analyze the impact of DTC on SRFS .

### 5.2 Spatial weight matrices and model specification

#### 5.2.1 Selection of spatial weight matrices

##### 5.2.1.1 Geographic distance weight matrix $W_{s}$

This study identifies the geographic coordinates ( lon , lat ) of the provincial capitals as reference points and calculates the great-circle distance $d_{\mathrm{ij}}$ between provinces i and j through Equation 15. The element in the corresponding position of the matrix $W_{s}$ is then determined by the inverse of the square of this distance (46), as shown in Equation 16:


$$d_{\mathrm{ij}}=\sqrt{{\left(\mathrm{lo}n_{i}-\mathrm{lo}n_{j}\right)}^{2}+{\left(\mathrm{la}t_{i}-\mathrm{la}t_{j}\right)}^{2}}$$ (15)

$$W_{s}=[\begin{array}{ccc} \frac{1}{{d_{1,1}}^{2}} & \ldots & \frac{1}{{d_{1,30}}^{2}}\\ \vdots & \ddots & \vdots \\ \frac{1}{{d_{30,1}}^{2}} & \ldots & \frac{1}{{d_{30,30}}^{2}} \end{array}]$$ (16)

##### 5.2.1.2 Economic and trade weight matrix $W_{e}$

Given the close relationship between the circulation digitalization and the interprovincial trade of agricultural products, data from the “C01” industry (i.e., agriculture, forestry, animal husbandry, and fishery) in the “China Regional Input-Output Table-2017” are extracted to form the matrix $W_{0}$ , where the element $w_{\mathrm{ij}}$ represents the quantity of agricultural products that province i supplies to province j . In terms of economic benefits, interprovincial trade of grain diversifies the income of the exporting regions and provides a time window for the importing regions to adjust their agricultural structures in response to external risks. In terms of environmental benefits, trade between grain-surplus and grain-deficit regions effectively addresses the overexploitation or waste of resources such as arable land. This indicates that both the import and export of grain are equally important for the development of the SRFS . Therefore, $w_{\mathrm{ij}}$ and $w_{\mathrm{ji}}$ are added together to represent the quantity of agricultural product trade between province i and province j , while the diagonal elements are set to 0 to construct the weight matrix of agricultural product trade $W_{e}$ , as shown in Equation 17:


$$W_{e}=W_{0}+{W_{0}}^{T}-2\mathrm{diag}(w_{1,1},w_{2,2},\ldots ,w_{30,30})$$ (17)

##### 5.2.1.3 Geography-trade nested weight matrix $W_{w}$

Using any single weight matrix to analyze spatial effects may introduce biases. To comprehensively consider both geographical distance and the intensity of agricultural product trade, we further construct a product-form geography-trade nested spatial weight matrix $W_{w}$ , where the element $w_{\mathrm{wij}}$ is as shown in Equation 18


$$w_{\mathrm{wij}}=w_{\mathrm{sij}}\times w_{\mathrm{eij}}$$ (18)

$w_{\mathrm{sij}}$ and $w_{\mathrm{eij}}$ are the elements of the normalized geographical distance weight matrix and the normalized trade weight matrix, respectively.

#### 5.2.2 Model selection

The selection of models is based on the results of the LM, LR, and Wald tests, which are presented in Table 7. Given the superiority of the Spatial Durbin Model (SDM) over the Spatial Lag Model (SRM) and the Spatial Autoregressive Model (SAR), the SDM is employed for analysis, while retaining the fixed-effects model.

**Table 7:** Model selection results.

Model type	Test	(1)	(2)	(3)
		$W_{s}$	$W_{e}$	$W_{w}$
SEM	LM	16.171 ***	20.397***	8.511***
	LR	57.50***	26.00***	43.25***
	Wald	60.16***	28.10***	50.50***
SAR	LM	37.078***	16.548 ***	16.281***
	LR	43.65***	28.92***	44.21***
	Wald	43.82***	32.72***	46.07***

Robust standard errors in parentheses ***p < 0.01, **p < 0.05, *p < 0.1.

### 5.3 Spatial econometric regression results

Table 8 presents the parameter estimation results of the spatial econometric models based on the three spatial weight matrices $W_{s}$ , $W_{e}$ , and $W_{w}$ . The regression results indicate the following: (1) In the geographical distance dimension, a 1% increase of DTC in neighboring regions is associated with an average 0.370% decrease in local SRFS , while the spatial autoregressive coefficient of SRFS is significantly positive. (2) In terms of trade interactions, 1% increase of DTC in regions with close trade ties is associated with an average 0.204% increase in local SRFS . This is because the influence of circulation digitalization can break through the geographical attenuation rule through cross-regional trade. (3) When considering both geographical distance and economic factors, the spatial spillover effect of circulation digitalization and the spatial autocorrelation effect of SRFS are both negative, but the significance is reduced compared to the first two estimates. This suggests that the resource competition in related regions and the synergistic effects in trade-related regions offset each other, weakening the impact of a single factor.

**Table 8:** SDM regression results.

Variables	$W_{s}$			$W_{e}$			$W_{w}$		
	(1)	(2)	(3)	(1)	(2)	(3)	(1)	(2)	(3)
	SDM	Direct	Indirect	SDM	Direct	Indirect	SDM	Direct	Indirect
DTC	0.381***	0.367***	−0.360**	0.325***	0.347***	0.401***	0.452***	0.413***	−0.361**
	(0.040)	(0.044)	(0.178)	(0.045)	(0.046)	(0.148)	(0.048)	(0.048)	(0.164)
Education	−0.145***	−0.155***	−0.210	−0.168***	−0.176***	−0.135	−0.158***	−0.186***	−0.241
	(0.034)	(0.037)	(0.186)	(0.040)	(0.040)	(0.105)	(0.034)	(0.030)	(0.151)
Employ	−0.002***	−0.003***	−0.011***	−0.004***	−0.004***	0.000	−0.003***	−0.003***	−0.008**
	(0.001)	(0.001)	(0.003)	(0.001)	(0.001)	(0.002)	(0.001)	(0.001)	(0.004)
Prim_prop	0.001	0.000	−0.007	0.008*	0.008	−0.005	0.003	0.005	0.014
	(0.005)	(0.005)	(0.014)	(0.005)	(0.005)	(0.014)	(0.005)	(0.004)	(0.024)
Invset	0.069*	0.072*	0.090	0.053	0.049	−0.074	0.021	−0.051	−0.646***
	(0.041)	(0.043)	(0.156)	(0.037)	(0.036)	(0.098)	(0.035)	(0.034)	(0.160)
Loan	−0.001	−0.000	0.011***	0.001	0.000	−0.012***	−0.000	0.000	0.007*
	(0.001)	(0.001)	(0.003)	(0.001)	(0.001)	(0.003)	(0.001)	(0.001)	(0.004)
W×Digit	−0.370***			0.204**			−0.385*		
	(0.116)			(0.090)			(0.223)		
rho	0.315***			0.289***			−0.342*		
	(0.094)			(0.069)			(0.205)		
sigma2_e	0.002***			0.002***			0.002***		
	(0.000)			(0.000)			(0.000)		
Fe	Yes	Yes	Yes	Yes	Yes	Yes	Yes	Yes	Yes
Observations	300	300	300	300	300	300	300	300	300
$R^{2}$	0.796	0.796	0.796	0.763	0.763	0.763	0.801	0.801	0.801

Robust standard errors in parentheses ***p < 0.01, **p < 0.05, *p < 0.1.

In terms of geographical distance, the direct effect of the DTC is significantly positive, while the indirect effect is significantly negative. That is, the improvement of DTC helps enhance local SRFS , but the “digital divide” can inhibit the development of surrounding areas through resource suction effects and market squeezing effects (such as monopolistic channels and price competition). In contrast, in terms of trade intensity, both the direct and indirect effects of DTC are significantly positive. This is because regions with close trade interactions in agricultural products often have long-term stable cooperative relationships, which efficiently adjust regional supply and demand imbalances of agricultural products, mitigate overexploitation or waste of resources, and balance economic and ecological benefits.

### 5.4 Analysis of mediating effect

The mechanism regression results are shown in Table 9. Model (1) and Model (2) present the regression results for FMD, while Model (3) and Model (4) present the regression results for industrial convergence (IC).

**Table 9:** Mediating effect results.

Variables	(1)	(2)	(3)	(4)
	FMD	SRFS	IC	SRFS
DTC	−0.787***	0.370***	0.007***	0.246***
	(0.085)	(0.065)	(0.003)	(0.061)
FMD		−0.053**		
		(0.021)		
IC				2.407**
				(1.043)
Controls	Yes	Yes	Yes	Yes
Fe	Yes	Yes	Yes	Yes
Observations	300	300	271	271
$\text{Adjusted}\;R^{2}$	0.677	0.779	0.589	0.779

Robust standard errors in parentheses ***p < 0.01, **p < 0.05, *p < 0.1. The T-value is enclosed in parentheses.

Combining Model (6) from Table 4 with Models (1), (2), (3), and (4) from Table 9 for mediating effect analysis reveals the following: (1) The result in Model (1) shows that the development of DTC significantly alleviates FMD, indicating that it can promote perfect competition and the convergence of prices to equilibrium levels by mitigating information asymmetry, thereby leveraging the price mechanism to adjust the factor market. The result in Model (2) demonstrates that both the increase in DTC and reduction in FMD can enhance SRFS , with the regression coefficient of DTC being lower than that in Model (7) of Table 4. It confirms that the digital transformation of the circulation can enhance SRFS by alleviating factor market distortion. (2) The result in Model (3) indicates that the development of DTC significantly promotes industrial convergence (IC). The digital transformation of the circulation, which integrates the entire chain of “production-processing-sales,” can break down the boundaries between traditional industries. The results in Model (4) show that both DTC and IC can simultaneously enhance SRFS , with the regression coefficient of DTC also being lower than that in Model (7) of Table 4. It suggests that industrial convergence is one of the transmission mechanisms through which DTC enhances SRFS . Hypothesis 2 is thus validated.

### 5.5 Heterogeneity analysis

#### 5.5.1 Heterogeneity in geographic location

To analyze the differences in the impact of DTC on SRFS across different geographic locations, the 30 provinces are divided into three sub-samples: eastern, central, and western regions. The regression results for each sub-sample are shown in Model (1) to Model (3) of Table 10. It can be observed that the DTC has a significant positive impact on SRFS across all geographic locations, but the magnitude of this impact varies significantly. The effect is the most pronounced in the central region, followed by the western region, and the least in the eastern region.

**Table 10:** Heterogeneity test results.

Variables	Geographic location			Functional orientation		
	(1)	(2)	(3)	(4)	(5)	(6)
	East	Middle	West	Produce	Sale	Produce_Sale
	SRFS	SRFS	SRFS	SRFS	SRFS	SRFS
DTC	0.282	1.142***	0.609***	0.689**	0.427***	0.641***
	(0.243)	(0.147)	(0.108)	(0.286)	(0.078)	(0.143)
Controls	Yes	Yes	Yes	Yes	Yes	Yes
Fe	Yes	Yes	Yes	Yes	Yes	Yes
Observations	100	60	110	130	70	100
$\text{Adjusted}\;R^{2}$	0.587	0.922	0.912	0.844	0.747	0.861

Robust standard errors in parentheses ***p < 0.01, **p < 0.05, *p < 0.1. The T-value is enclosed in parentheses.

This variation may be attributed to the following reasons: (1) In terms of factor market distortion, circulation digitalization alleviates information asymmetry and corrects factor distortions in the central region, thereby significantly enhancing SRFS . However, in the western region, geographical and other constraints limit the theoretical impact of DTC . Meanwhile, the eastern region already has a relatively well-developed factor allocation, resulting in a smaller marginal utility from DTC . (2) Regarding industrial convergence, circulation digitalization can effectively improve the relatively poor industrial convergence foundation in the central region, and interprovincial trade in agricultural products helps resolve the contradiction between smallholder economies and large market demands. In contrast, the industrial structure in the western region is relatively single, and the role of digital circulation is more manifested in the partial transformation of existing industries. The eastern region already has a high degree of industrial convergence, making it more difficult for DTC to further promote industrial convergence and structural upgrading in this area.

#### 5.5.2 Heterogeneity in functional orientation

To compare the impact of digitalization on SRFS across regions with different food functional orientations, the 30 provinces are divided into three sub-samples: major grain-producing areas, major grain-consuming areas, and balanced production and consumption areas. The regression results for each sub-sample are shown in Model (4) to Model (6) of Table 10. The results show that for different functional orientations, the DTC is significantly positively correlated with SRFS , but the effect is significantly higher in major grain-producing areas than in major grain-consuming areas and balanced production and consumption areas.

This variation may be attributed to the following reasons: (1) In terms of factor market distortion, major grain-producing areas have larger agricultural production scales, which can fully leverage the scale effect advantages of DTC in correcting factor distortions such as labor aging and insufficient capital investment. In contrast, major grain-consuming areas and balanced areas have higher degrees of factor marketization and relatively optimized factor allocation, resulting in lower marginal benefits from circulation digitalization. (2) Regarding industrial convergence, major grain-producing areas generally have shorter industrial chains, and DTC can fully unleash the potential and substantial benefits of industrial convergence. In contrast, major grain-consuming areas and balanced areas have more mature industrial convergence, where digitalization mainly serves to optimize circulation efficiency and has limited impact on industrial structure upgrading, facing the ceiling constraint of inventory optimization.

### 5.6 Threshold effect analysis

This study employs the Bootstrap resampling method, conducting 1,000 replications for both single-threshold and double-threshold tests. The results are presented in Table 11. It is found that the single-threshold models for the threshold variables DTC and AIA both pass the significance test, while the double-threshold models fail to meet the significance criteria. Therefore, the single-threshold model is selected to examine the nonlinear effect of the DTC and the threshold effect of agricultural industrial agglomeration ( AIA ) on the impact of DTC on SRFS . The threshold values are determined to be 0.3562 for DTC and 0.4282 for AIA.

**Table 11:** Threshold effect test.

Models	Threshold variables	Model type	*F*	*p*	BS	Critical value			Threshold estimate
						10%	5%	1%	
(1)	DTC	Single	34.60	0.008	1,000	21.204	26.035	34.099	0.3562
		Double	4.23	0.851	1,000	21.266	32.886	60.861	–
(2)	AIA	Single	64.01	0.008	1,000	26.078	31.368	54.792	0.4282
		Double	8.87	0.630	1,000	23.063	28.093	45.433	–

The regression results of the single-threshold models with DTC and AIA as threshold variables are shown in Model (1) and Model (2) of Table 12.

The results in Table 12 show the following: (1) Regardless of the level of DTC and the degree of AIA , the impact of DTC on SRFS remains significantly positive, further confirming the robustness of the benchmark regression. 2 The promoting effect of DTC on SRFS exhibits diminishing marginal utility. That is, the effect is significantly greater when DTC<0.3562 than when DTC>0.3562 . When the level of DTC is high, further increases in digitalization cannot cover other contradictions in the process of enhancing SRFS , leading to a continuous decline in marginal utility. (3) Under conditions of high agricultural industrial agglomeration ( AIA>0.4282 ), the promoting effect of DTC on SRFS is greater, while under conditions of low agglomeration ( AIA<0.498 ), the effect is significantly reduced. This divergence is essentially the spatial mapping of the increasing returns to scale of the digital economy: in areas with high industrial agglomeration, the scale effects of resources and factors reduce the friction coefficient of technology diffusion, achieving a nonlinear leap in the effect of DTC . In contrast, in areas with low agglomeration, the dispersion of resources and factors leads to rigid marginal costs of digital transformation, making it difficult to break through the threshold of economies of scale, and thus the promoting effect is significantly weakened. Therefore, Hypothesis 3 is validated.

**Table 12:** Threshold model results.

Variables	(1)	(2)
	SRFS	SRFS
DTC×I(Threshold≤ω)	0.528***	0.255***
	(0.035)	(0.041)
DTC×I(Threshold>ω)	0.401***	0.498***
	(0.035)	(0.032)
Controls	Yes	Yes
$\text{Adjusted}\;R^{2}$	0.762	0.723

Robust standard errors in parentheses ***p < 0.01, **p < 0.05, *p < 0.1. The T-value is enclosed in parentheses.

## 6 Conclusion and discussion

With rapid population growth and intensifying environmental pressures, SRFS has garnered widespread attention worldwide. The circulation industry is a crucial foundation of the food system. With the application of the Internet of Things and big data, digital transformation in the circulation industry is accelerating and may reshape the structure of the food industry. Existing studies have explored the digital economy and food systems extensively. However, most of them focus on the digital transformation of agricultural production or the macro-level effects of the overall digital economy on the economic and environmental performance of food systems (30, 55, 56). In comparison, the connecting role of the circulation sector across production, supply, and sales has received less attention, particularly from the perspective of circulation digitalization.

Using panel data from 30 Chinese provinces (2013–2022) and a fixed-effects model, we find that DTC positively affects SRFS. We also identify a positive moderating effect of government financial support related to agriculture in this relationship. These findings offer evidence that circulation digitalization can contribute to long-term food security and enrich research on the digital economy and food systems at a more specific sectoral level.

Considering the spatial spillovers of digital technologies (47), we construct three spatial weight matrices from the perspectives of geographical distance and agricultural trade for spatial econometric analysis. The results show clear heterogeneity in indirect effects across spatial interaction channels. Under geographical distance weights, DTC has a significant negative indirect effect on SRFS, implying an inhibitory effect on SRFS in neighboring regions. This pattern is consistent with the siphon effect and the digital divide observed in practice. Under trade-intensity weights, however, the indirect effect becomes positive. This suggests that DTC can enhance SRFS in regions with close trade ties by alleviating regional mismatches in agricultural product supply and demand. Overall, these results highlight the role of digital technology in reducing circulation barriers and improving resource allocation efficiency across regions.

Prior research indicates that the digital economy can alleviate factor misallocation (26) and catalytic industrial structure upgrading (51), which in turn supports agricultural resilience and green production. Building on this line of reasoning, we introduce factor market distortion and industrial convergence as mediating variables. The results suggest that DTC can enhance SRFS by alleviating factor market distortion and promoting the integration of primary, secondary, and tertiary industries.

Industrial agglomeration has become an important engine of regional development in countries such as China, and regions with higher agglomeration tend to display stronger economic activity (40). Accordingly, we use agricultural industrial agglomeration as a threshold variable to examine the single-threshold effect of DTC on SRFS. In addition, motivated by the law of diminishing returns, we further explore the nonlinear relationship between DTC and SRFS. These findings extend the understanding of how digital circulation affects food system resilience under different industrial foundations and provide a basis for more differentiated digital policies.

Compared with existing research, our results are broadly consistent with the prevailing conclusion that the digital economy supports food security and sustainable agricultural development. For example, data elements (49) and the overall development level of the digital economy (50) have been found to promote sustainable agricultural development. This study shifts the focus to the circulation industry, a key link in the food system, and provides evidence that its digital transformation contributes positively to SRFS, while also clarifying mechanisms and heterogeneity.

Meanwhile, we also observe patterns that are not fully aligned with some existing findings. Many studies emphasize positive spatial spillover effects of the digital economy on sustainable agricultural development in neighboring areas (8, 53). In contrast, our results indicate that the spillover effects of digital circulation are spatially heterogeneous: the indirect effect is negative for geographically adjacent areas but positive for regions with close agricultural product trade ties. A plausible explanation is that circulation digitalization can weaken traditional distance constraints for perishable agricultural products (e.g., vegetables and fruits). As a result, locally produced products are no longer primarily circulated within nearby areas, and cross-regional allocation becomes more feasible. This shift may improve sustainability in distant areas with strong trade connections, while making siphon effects on geographically adjacent areas more pronounced.

Based on the findings, we propose the following recommendations. (1) Strengthen rural digital infrastructure, such as 5G networks and smart warehousing, to improve cross-regional agricultural circulation. Encourage the adoption of digital technologies such as e-commerce and intelligent warehousing to enhance the quality and efficiency of cross-regional circulation. (2) Tailor policy strategies to local conditions—enhance factor markets and industrial foundations in less developed western regions, while paying greater attention to external constraints and coordination challenges in eastern regions and major grain-consuming areas. (3) Promote agricultural industrial agglomeration by planning industry parks and clusters to realize scale and synergy effects, thereby creating a more supportive environment for digital circulation.

However, this study has limitations. First, we focus on the unidirectional impact of DTC on SRFS and do not explicitly examine potential bidirectional dynamics. Improvements in SRFS may also promote DTC, forming feedback effects. Future research could apply bidirectional fixed-effects models or simultaneous equations models to examine potential two-way causal relationships. Second, we do not consider heterogeneous impacts under extreme scenarios (e.g., natural disasters, public health events, or international food price fluctuations). Such shocks may change food system resilience and may also alter the effects of circulation digitalization. Future work could introduce scenario dummies to assess whether the effects differ across shock conditions, improving the realism and policy relevance of the conclusions.

## Data Availability

The original contributions presented in the study are included in the article/supplementary material, further inquiries can be directed to the corresponding authors.
