# Correction: Digitalization of circulation and sustainable resilience of food systems: spatial effects and transmission mechanisms

**DOI:** 10.3389/fnut.2026.1832418

**Published:** 2026-04-02

**Authors:** Hengli Wang, Qi Wang, Xinpeng Cao, Zuojunli Zhang

**Affiliations:** 1 Wuhan University of Cyber Security Preparatory Office, Wuhan, China; 2 School of Mathematics and Statistics, Zhongnan University of Economics and Law, Wuhan, China; 3 Wenlan School of Business, Zhongnan University of Economics and Law, Wuhan, China; 4 Independent Researcher, Wuhan, China

**Keywords:** digital circulation, factor market distortions, food security, food systems, industrial convergence, sustainable resilience

Affiliation for the author, Zuojunli Zhang, was erroneously provided as, “Faculty of Education, Universiti Kebangsaan Malaysia, Bangi, Selangor, Malaysia”. Their correct affiliation should read as, “Independent Researcher, Wuhan, China.” Author [Duochenxi Liu] was erroneously included as an author in the published article. The correct author list reads:

Hengli Wang^1*^, Qi Wang^2^, Xinpeng Cao^3^ and Zuojunli Zhang^4*†^ ^1^Wuhan University of Cyber Security Preparatory Office, Wuhan, China ^2^School of Mathematics and Statistics, Zhongnan University of Economics and Law, Wuhan, China ^3^Wenlan School of Business, Zhongnan University of Economics and Law, Wuhan, China ^4^Independent Researcher, Wuhan, China

The author contributions statement was erroneously given as “HW: Conceptualization, Data curation, Funding acquisition, Resources, Software, Validation, Visualization, Writing – original draft, Writing – review & editing. QW: Conceptualization, Data curation, Funding acquisition, Investigation, Methodology, Software, Supervision, Visualization, Writing – original draft, Writing – review & editing. XC: Conceptualization, Data curation, Formal analysis, Resources, Software, Visualization, Writing – original draft, Writing – review & editing. ZZ: Conceptualization, Resources, Validation, Writing – original draft, Writing – review & editing. DL: Validation, Visualization, Writing – original draft, Writing – review & editing.” The correct author contributions statement is “HW: Conceptualization, Data curation, Funding acquisition, Resources, Software, Validation, Visualization, Writing – original draft, Writing – review & editing. QW: Conceptualization, Data curation, Funding acquisition, Investigation, Methodology, Software, Supervision, Visualization, Writing – original draft, Writing – review & editing. XC: Conceptualization, Data curation, Formal analysis, Resources, Software, Visualization, Writing – original draft, Writing – review & editing. ZZ: Conceptualization, Resources, Validation, Formal analysis, Data curation, Writing – original draft, Writing – review & editing.”

Author Duochenxi Liu was erroneously assigned as corresponding author. The correct corresponding author is Zuojunli Zhang.

The original version of this article has been updated.

